# Editorial: Macrophage activation syndrome in children in the era of COVID-19

**DOI:** 10.3389/fped.2023.1222522

**Published:** 2023-06-14

**Authors:** Oksana Boyarchuk, Alla Volokha

**Affiliations:** ^1^ Department of Children's Diseases and Pediatric Surgery, I.Horbachevsky Ternopil National Medical University, Ternopil, Ukraine; ^2^ Department of Pediatrics, Pediatric Infectious Diseases, Immunology and Allergology, Shupyk National Healthcare University of Ukraine, Kyiv, Ukraine

**Keywords:** macrophage activation syndrome, MAS, secondary hemophagocytic lymphohistocytosis, sHLH, COVID-19, multisystem inflammatory syndrome in children, MIS-C, inborn errors of immunity

**Editorial on the Research Topic** Macrophage activation syndrome in children in the era of COVID-19

Until now, SARS-CoV-2 infection has caused the death of about 7 million people worldwide. Although children commonly have a mild course of COVID-19, some of them present with severe hyperinflammation, multisystem inflammatory syndrome (MIS), and even macrophage activation syndrome (MAS), which causes multiple organ damage and poor prognosis. MAS, also known as secondary hemophagocytic lymphohistiocytosis (HLH), is a life-threatening complication of various inflammatory disorders. Primary HLH is caused by certain genetic variants that determine defects of cytotoxic lymphocytes. Secondary or acquired forms of HLH may be caused by infections, malignancy, underlying inflammation, or drugs. The term MAS is often used with rheumatic diseases, particularly with systemic juvenile idiopathic arthritis. However, it is also used in patients with juvenile systemic lupus erythematous, Kawasaki disease (KD), etc. Whereas the clinical manifestations of multisystem inflammatory syndrome in children (MIS-C) are similar to the symptoms of Kawasaki disease, it is described as Kawasaki-like syndrome related to SARS-CoV-2 infection, which makes it possible to use the term MAS for this disease caused by COVID-19. However, it is not completely clear whether the pathogenesis of the development of MIS-MAS and KD-MAS is the same. In general, the line between primary and secondary HLH is also very blurred, as the same factors can cause activation in patients with primary HLH, and genetic abnormalities can occur in the secondary syndrome.

Frequently, it is very difficult to distinguish MAS from other complications. A high percentage of MAS cases are not diagnosed early, leading to a poor prognosis and high mortality.

The causes of MAS in patients with severe COVID-19, post-COVID conditions are not completely clear. The innate immune system plays a central role in the initiation of MAS. Genetic predisposition and possible triggers on the background of active inflammation cause an increase in macrophage and T-lymphocyte activity, leading to a cytokine storm that can result in tissue damage, multiorgan dysfunction, and even death. The definition of predictive factors for developing MAS in patients with MIS-C and severe COVID-19 is very important.

This research study aimed to present the clinical phenotypes and outcomes of MAS in children and define the risk factors for the development of MAS in patients with MIS-C and Kawasaki-like disease on the background of SARS-CoV-2 infection.

This research study collected four articles from different countries. Buda et al. analyzed data from the Polish MIS-C registry. Fifty-nine children among 270 patients with MIS-C fulfilled MAS classification criteria. Using multivariable analysis, the authors evaluated clinical and laboratory parameters to determine risk factors for MAS in patients with MIS-C. Age, atypical Kawasaki disease, skin erosions, low lymphocyte and platelet counts, albumin and sodium levels, high C-reactive protein, procalcitonin, ferritin, D-dimers, triglycerides, serum creatinine, urea, *γ*-glutamyl transpeptidase, and neutrophil count were found as significant factors associated with MAS in MIS-C patients. High procalcitonin, ferritin, and fibrinogen levels were risk factors for MAS at admission.

Hua-Yong Zhang et al. conducted a retrospective case–control study investigating the early predictive factors for Kawasaki disease complicated by MAS. Using multivariate analysis, platelets and serum ferritin were defined as independent early predictive factors for developing MAS in children with KD. The authors figured out that splenomegaly, hypoproteinemia, hypofibrinogenemia, high levels of ferritin, aspartate aminotransferase, alanine aminotransferase, and lactate dehydrogenase on admission; an increase in ferritin and triglycerides levels and a decrease in hemoglobin levels after the first dose of intravenous hemoglobin (IVIG) treatment; and IVIG resistance can be possible predictors of MAS development in patients with KD.

Boyarchuk et al. presented a clinical case of virus-induced HLH in a patient with inborn errors of immunity, autoimmune polyendocrinopathy-candidiasisectodermal dystrophy (APECED). The patient developed retinopathy with macular atrophy and autoimmune hepatitis after the first episode of SARS-CoV-2 infection. Epstein–Barr virus infection and a new episode of COVID-19 with pneumonia triggered the development of HLH with a fatal outcome. This study confirms that the boundary between primary and secondary HLH is conditional since viruses were the cause of the development of HLH in a child with a genetic defect. The reported case was the first that described the development of HLH in patients with APECED.

The first-line treatment of KD, MIS-C, and MAS is similar and includes IVIG and/or high dose of corticosteroids. Etoposide and biological agents are used as the second-line therapy. In the research of Buda et al., none of the patients with MIS-C/MAS required second-line therapy. Vaccination against SARS-CoV-2 is considered an effective strategy for preventing the severe course of COVID-19, including MIS-C and MAS/HLH. A study by Consolini et al. described a case of MIS-C with significant hepatic and pancreatic dysfunction that developed in adolescents vaccinated against SARS-CoV-2 after contact with a COVID-19-positive patient 10 days before admission. The authors discussed improving the definition of MIS-C and defining the risk factors for developing MIS-C in vaccinated patients.

The contributors to this special topic underline the difficulties in diagnosing MAS in patients with COVID-19, MIS-C, and KD. It shares overlapping clinical and laboratory features with other conditions in patients with COVID-19, accompanied by hyperinflammation and cytokine storm. More attention should be paid to this serious and life-threatening complication in the era of COVID-19 to improve its recognition. Predictive factors for the development of MAS in patients with MIS-C, KD, and COVID-19 determined in the studies of this topic can allow the administration of timely and appropriate therapy and prevent serious consequences. The editors hope that this collection answers difficult questions and encourages further research on this topic.

